# A novel NHEJ gene signature based model for risk stratification and prognosis prediction in hepatocellular carcinoma

**DOI:** 10.1186/s12935-023-02907-9

**Published:** 2023-04-04

**Authors:** Zhu Lin, Zhenkun Huang, Yunxing Shi, Yichuan Yuan, Yi Niu, Binkui Li, Yunfei Yuan, Jiliang Qiu

**Affiliations:** 1 State Key Laboratory of Oncology in South China and Collaborative Innovation Center for Cancer Medicine, Sun Yat-Sen University Cancer Center, Guangzhou, China; 2 Department of Liver Surgery, Sun Yat-Sen University Cancer Center, 651 Dongfeng Road East, Guangzhou, 510060 People’s Republic of China

**Keywords:** Hepatocellular carcinoma, Non-homologous DNA end joining, Immune infiltrated landscape, Prognostic model, Risk stratification

## Abstract

**Background:**

Non-homologous DNA end joining (NHEJ) is the predominant DNA double-strand break (DSB) repair pathway in human. However, the relationship between NHEJ pathway and hepatocellular carcinoma (HCC) is unclear. We aimed to explore the potential prognostic role of NHEJ genes and to develop an NHEJ-based prognosis signature for HCC.

**Methods:**

Two cohorts from public database were incorporated into this study. The Kaplan–Meier curve, the Least absolute shrinkage and selection operator (LASSO) regression analysis, and Cox analyses were implemented to determine the prognostic genes. A NHEJ-related risk model was created and verified by independent cohorts. We derived enriched pathways between the high- and low-risk groups using Gene Set Enrichment Analysis (GSEA). CIBERSORT and microenvironment cell populations-counter algorithm were used to perform immune infiltration analysis. *XRCC6* is a core NHEJ gene and immunohistochemistry (IHC) was further performed to elucidate the prognostic impact. In vitro proliferation assays were conducted to investigate the specific effect of *XRCC6*.

**Results:**

A novel NHEJ-related risk model was developed based on 6 NHEJ genes and patients were divided into distinct risk groups according to the risk score. The high-risk group had a poorer survival than those in the low-risk group (*P* < 0.001). Meanwhile, an obvious discrepancy in the landscape of the immune microenvironment also indicated that distinct immune status might be a potential determinant affecting prognosis as well as immunotherapy reactiveness. High *XRCC6* expression level associates with poor outcome in HCC. Moreover, *XRCC6* could promote HCC cell proliferation in vitro.

**Conclusions:**

In brief, this work reveals a novel NHEJ-related risk signature for prognostic evaluation of HCC patients, which may be a potential biomarker of HCC immunotherapy.

**Supplementary Information:**

The online version contains supplementary material available at 10.1186/s12935-023-02907-9.

## Introduction

Globally ranking the fourth leading cause of cancer- related death, hepatocellular carcinoma (HCC) is a malignant disease with poor prognosis [1]. Despite continuous progress in diagnosis and treatment, the 5-year survival rate of HCC remains poor due to the spread, metastases and high rate of recurrence. Several studies aimed to construct effective predictive models in HCC previously, including deep learning-based multi-omics model, radiomics model and gene signature models [2–6]. Studies have proved that m6A-related genes, ferroptosis-related genes, and aging-related genes were all associated with cancer prognosis [7, 8]. However, it is still hard to predict patient’s prognosis effectively, highlighting the need to identify HCC biomarkers.

Non-homologous DNA end joining (NHEJ) is the predominant DNA double-strand break (DSB) repair pathway in mammalian cells. NHEJ is also important for B cell and T cell development. Mutation or absence of NHEJ can result in immunodeficiency [9]. Several studies have reported that core NHEJ factors are overexpressed in certain tumor tissues. The dysregulation or hyperactivation of NHEJ machinery has been linked to cancer and resistance to anti-tumor treatment [10]. As such, NHEJ components have emerged as drug targets for cancer therapy [11], and DNA-dependent protein kinase (DNA-PK) inhibitors have entered clinical trials. DNA damage repair factors, like *XRCC6, XRCC5, PARP1*, were crucial and closely related to cancer development and progression [12, 13]. Whether NHEJ factors-based model is effective for HCC prediction is still unclear and worth to be revealed.

Since that there were only 20% of HCC patients which exhibited response to PD-1/PD-L1 antibody, patients’ stratification and selection is crucial and meaningful [14]. The efficacy of immunotherapy is partly dependent on immune infiltration, especially cytotoxic T cells [15]. Therefore, to identify patients who would benefit from immunotherapy, prediction of immune infiltration is favorable. Here, we also aimed to understand the correlation between NHEJ genes and immune infiltration in HCC.

In this study, we identified six prognosis-related NHEJ factors using the LASSO methods, on basis of which, we subsequently constructed a predictive model for HCC patients. We first used data from The Cancer Genome Atlas (TCGA) to construct an NHEJ-based signature associated with the survival rates of liver hepatocellular carcinoma (LIHC) patients. One of large data from the Gene Expression Omnibus (GEO) ,GSE14520, was then used to validate the predictive ability of this signature. Our model was proved to be effective to predict prognosis of HCC in two independent cohorts. In addition, The NHEJ-related model was confirmed to be associated with tumor immune infiltration and might be used to predict immunotherapy response of HCC.

## Methods and materials

### Patient data collection

The RNA-seq transcriptome data and clinical information of LIHC patients were extracted from TCGA (https://tcga-data.nci.nih.gov/tcga/) and GSE14520 databases (https://www.ncbi.nlm.nih.gov/geo/). Patients with missing survival data or overall survival (OS) < 30 days, or without definitive histopathological diagnosis were excluded. Patients with OS < 30 days were excluded from analysis, as these patients may have had too advanced disease or complications of treatments. The TCGA dataset (n = 356) served as a training cohort. The GSE14520 dataset (n = 225) was used as the validation cohort. The RNA-seq transcriptome data of TCGA dataset were downloaded in the format of fragments per kilobase of exon model per million mapped reads (FPKM) normalized. The count data of expression array from GSE14520 were acquired by “GEOquery” package. The different gene expression datasets were normalized using the “limma” and “SVA” R packages to remove the potential batch effect. NHEJ-related genes, shown in Supplementary Table S1, were selected and downloaded from hallmark gene sets in the Molecular Signatures Database (MSigDB).

### Human HCC samples

The samples of HCC and the paired adjacent normal tissues were obtained from surgical resection at Sun Yat-sen Cancer Center (n = 175, from January 2013 to June 2015). All patients have pathology confirmed diagnosis of HCC. Patients with missing survival data or overall survival (OS) < 30 days were excluded. The tissue microarray was constructed containing a total of 175 pairs of HCC samples and matched adjacent normal tissues. Paired data were analyzed by paired *t*-test.

### Construction and validation of the NHEJ signature

Thirteen NHEJ genes were first subjected to univariate Cox regression analysis (p < 0.05). Following this, the LASSO regression analysis was performed to narrow down the prognostically significant candidate NHEJ genes. Then, multivariate Cox regression analysis was used to determine the best weighting coefficient of each prognostically significant candidate NHEJ genes. The risk score was calculated using the following the equation according to the literature [16]: Risk score = $\sum (\mathrm{expression level of each target gene }\times \mathrm{ corresponding coefficients})$.

According to the cut-off point of risk scores derived from maximally selected log-rank statistics, LIHC patients in the TCGA training cohort were divided into low and high-risk groups. The Kaplan–Meier method was utilized to estimate OS and the log-rank test was used to compare the differences of OS between the two groups.

To validate the NHEJ signature, the risk score of HCC cases in the GSE14520 dataset were calculated using the same formula as the TCGA cohort. Cases in the validation set were also divided into two groups according to the cut-off point of risk score obtained from the maximally selected log-rank statistics. Survival curves of the low- and the high-risk groups in the validation cohort were also estimated using the Kaplan–Meier method and were compared via the log-rank test.

### Functional enrichment analysis

To investigate the potential molecular mechanisms of the NHEJ signature, GSEA were performed in the TCGA and GSE14520 datasets. The analyses of perform Genetic Ontology (GO) term and Kyoto Encyclopedia of Genes and Genomes (KEGG) pathway were conducted by GSEA 4.0.1 software. After 1000 permutations, significant enrichment was defined as the pathway with the value of false discovery rate (FDR) < 0.25 and normalized p < 0.05.

### Evaluation of the immune landscape

The infiltrating immune cells levels were calculated by CIBERSORT [17] and microenvironment cell populations-counter (MCP)-counter algorithms [18] in each HCC sample and compared between the high-score and low-score groups. The Mann–Whitney *U* test was performed to compare the differential expression levels of *PDCD1, CD274, PDCD1LG2* and *CTLA4* between the two risk groups.

### Establishment of a predictive nomogram based on the NHEJ signature

Using the TCGA training set, a nomogram integrating the NHEJ signature and clinical stage to predict individual survival was established. In addition, calibration curves and the area under the curve (AUC) for the OS probability at 1, 3, 5 years were plotted to evaluate the predictive accuracy of this nomogram in the TCGA set and the GEO validation set.

Additional information is provided in Additional file 1: Methods S1.

### Statistical analysis

Continuous data are shown as the mean ± standard deviation (SD) and were compared using Student’s *t*-test. Categorical variables were analyzed using the chi-square (*χ*^2^) test. Cox regression analyses were performed to determine the significantly independent prognostic factors for OS. A prognostic nomogram model was established using the “rms” R package, while its predictive accuracy was assessed via the creation of calibration curves. Statistical analysis was performed using SPSS (version 22.0) and R software (version 4.0.1). The threshold of statistical significance was set at a p-value < 0.05.

## Results

### Identification of prognostic NHEJ genes

**Fig. 1:**
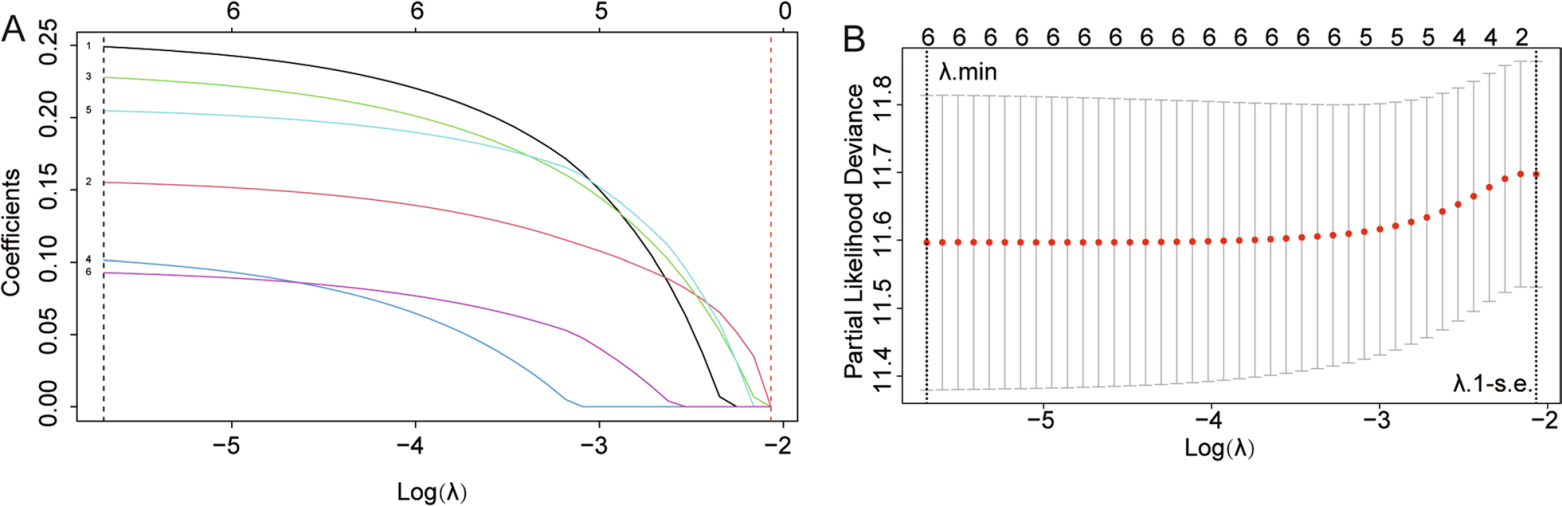
Identification of a prognosis-related NHEJ-based signature in the TCGA training cohort. **A** LASSO coefficients of prognosis-associated NHEJ genes, each curve represents a gene. **B** Selection of the optimal candidate genes in the LASSO model. The two dotted vertical lines were drawn at the optimal scores by λ.min criteria and 1-s.e. criteria (At λ.min criteria including all the six genes)

As represented in the flowchart (Additional file 1: Fig. S1), our study focused on the NHEJ pathway genes. After excluding 31 cases with unsatisfied follow-up or those lacking important clinical information, 343 cases from the TCGA training set were included to identify prognosis-related NHEJ genes. In addition, 229 cases from the GSE14520 dataset were used as verification cohort. Six NHEJ genes, *DCLRE1C, FEN1, PRKDC, XRCC4, XRCC5* and *XRCC6*, were identified to be associated with HCC prognosis using univariate Cox regression (Table 1). All the six genes were negatively correlated with OS of HCC patients, indicating that NHEJ genes might act as oncogenes in HCC. Then the six genes were subjected to LASSO Cox regression, with a significant correlation between and OS at minimum values (Fig. 1A). Further disciplinary regression was performed to take λ.min criteria as independent risk factors for prognosis in patients with HCC (Fig. 1B). Finally, a six-NHEJ risk signature was derived according to 343 LIHC cases in the TCGA dataset, whose risk score was specifically calculated based on a linear combination of gene expression levels and their corresponding regression coefficients from the multivariate Cox analysis. The specific formula was as follows: Risk score = *DCLRE1C* × 0.249100386163019 + *FEN1* × 0.155181162762813 + *PRKDC* × 0.227886948457229 + *XRCC4* × 0.101357794279081 + *XRCC5* × 0.204722206320064 + *XRCC6* × 0.0928527312741607.

**Table 1:** Identification of prognostic NHEJ genes using univariate Cox regression

Gene Symbol	P value	Hazard ratio
*DCLRE1C*	< 0.001	1.999(1.390−2.875)
*FEN1*	< 0.001	1.490(1.224−1.813)
*PRKDC*	< 0.001	1.630(1.284−2.070)
*XRCC4*	0.004	1.590(1.162−2.177)
*XRCC5*	< 0.001	2.039(1.432−2.902)
*XRCC6*	< 0.001	1.721(1.267−2.338)

### Prognostic Value of the NHEJ Signature in the Training Cohort

**Fig. 2:**
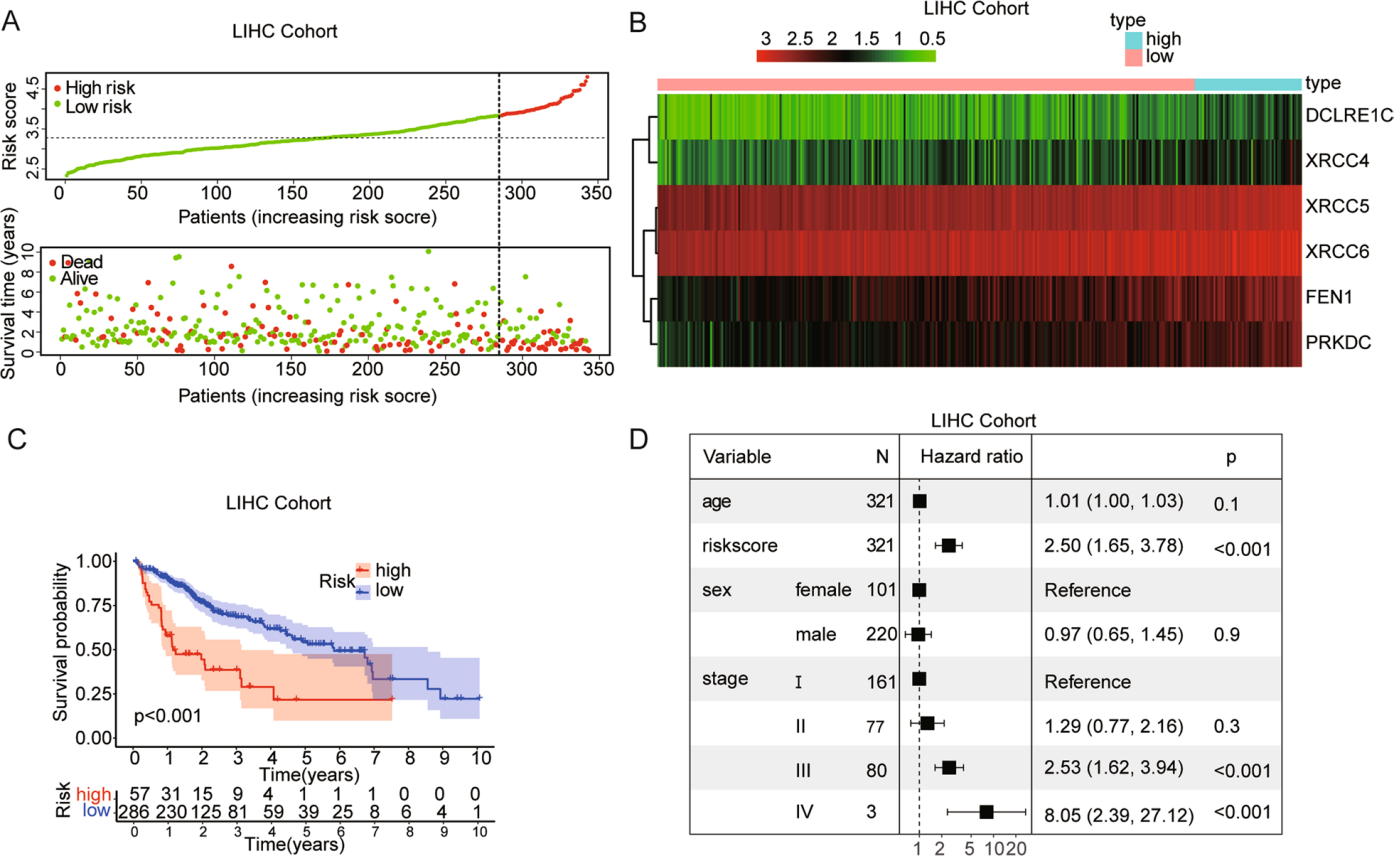
Assessment of prognostic value of the NHEJ signature model in the TCGA training cohort. **A**. The risk score, survival time and survival status in the training cohort. **B** The heatmap showing expression profiles of the 6 NHEJ genes. **C** Kaplan–Meier curves for the OS of patients in the high- and low-risk group. **D** Multivariate Cox regression analysis of NHEJ genes signature and other clinicopathological factors in the training cohort

The cut-off value of risk scores was determined as 3.84 using the maximally selected log-rank statistics in the TCGA training cohort (Additional file 1: Fig. S2A) to divide the cases into low-risk and high-risk groups. The distribution of the risk score showed that more death events were observed in the high-risk group (Fig. 2A). We found that all six genes were significantly up-regulated in the high-risk group, which was consistent with their prognostic value (Fig. 2B). In the TCGA cohort, the Kaplan–Meier curve suggested that the OS of patients in the low-risk group was significantly longer than that of patients in the high-risk group (p < 0.001; Fig. 2C). Figure 2D showed the results of multivariate Cox regression analysis (HR = 2.50, 95% CI = 1.65–3.78, p < 0.001).

### Prognostic validation of the NHEJ signature in GSE14520 dataset

**Fig. 3:**
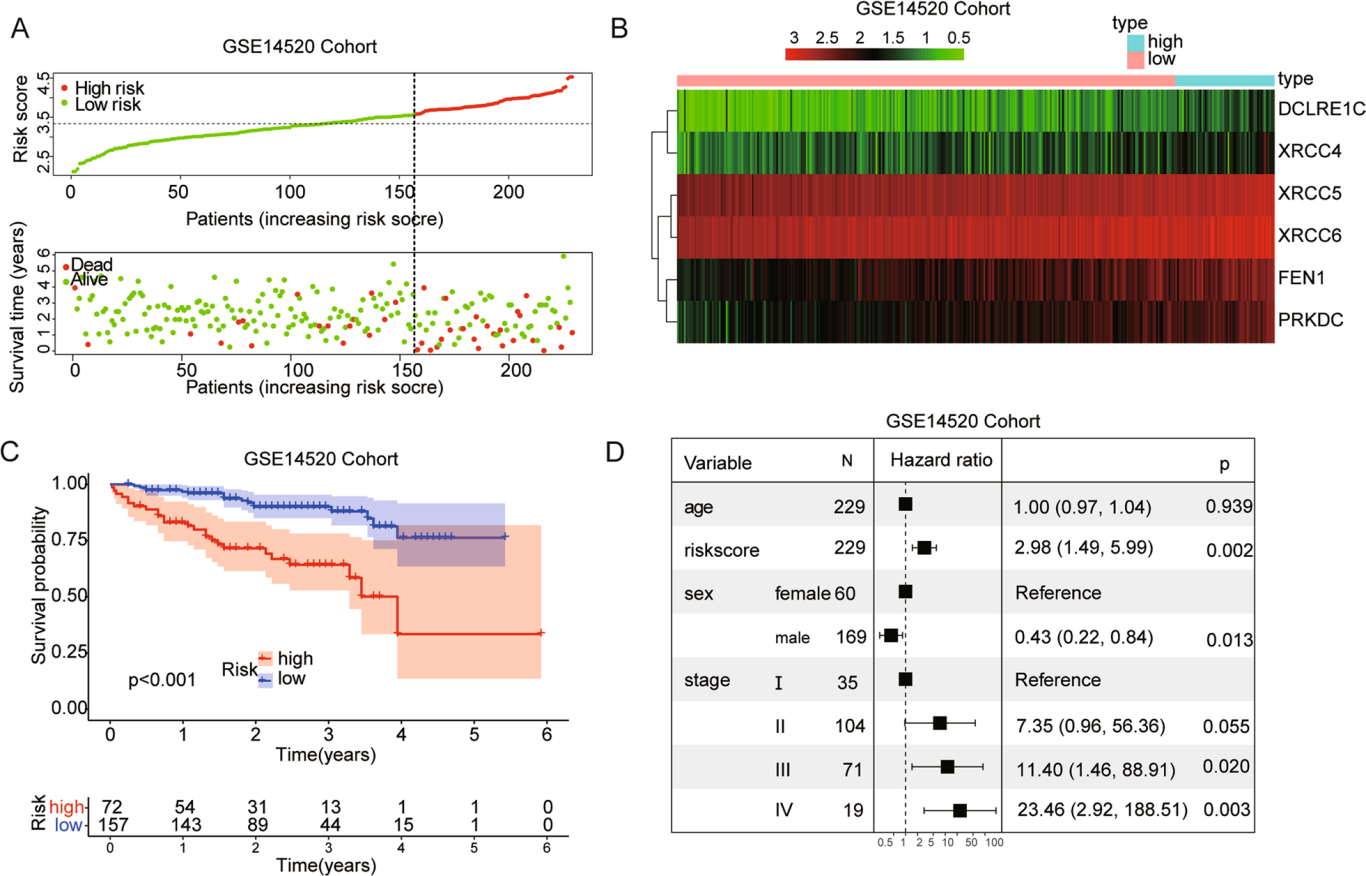
Assessment of prognostic value of the NHEJ signature model in the GSE14520 validation cohort. **A** The risk score, survival time and survival status in the validation cohort. **B** The heatmap showing expression profiles of the 6 NHEJ genes. **C** Kaplan–Meier curves for the OS of patients in the high- and low-risk group. **D** Multivariate Cox regression analysis of NHEJ genes signature and other clinicopathological factors in the validation cohort

According to the risk score based on the maximally selected log-rank statistics, 229 cases were divided into the high- and low-risk groups in the GSE14520 validation cohort (Additional file 1: Fig. S2B). The distribution of the risk score showed that more death events were observed in the high-risk group (Fig. 3A). The six genes were also significantly up-regulated in the high-risk group in the GSE14520 cohort (Fig. 3B). The Kaplan–Meier curve suggested that the OS of patients in the low-risk group was significantly longer than that of patients in the high-risk group (p < 0.001; Fig. 3C). Figure 3D showed the results of multivariate Cox regression analysis (HR = 2.98, 95% CI = 1.49–5.99, p = 0.002).

### Functional enrichment analysis

We then performed the GSEA to verify differential pathways between low and high-risk group in order to investigate the underlying functional mechanism. In the high-risk group, KEGG enrichment analysis found that genes were primarily enriched in DNA replication, mismatch repair, homologous recombination, cell cycle, non-homologous end joining in both datasets. By contrast, multiple metabolism pathways were enriched in low-risk group mainly (Table 2, and Additional file 1: Fig. S3). GO enrichment analysis showed similar results and found that genes were primarily enriched in cell cycle DNA replication, double strand break repair in the high-risk group, while multiple metabolism pathways were enriched in low-risk group mainly (Additional file 1: Fig. S3). Full GSEA results are available in Additional file 2: Table S4 and Additional file 3: Table S5.

**Table 2:** KEGG enrichment analysis between the high- and low-risk subgroups in TCGA training cohort and the GEO validation cohort

KEGG ID	ES	p-value	FDR
TCGA cohort			
DNA replication	− 0.8061	0.002	0.0407
Mismatch repair	− 0.7773	0.0019	0.0458
Homologous recombination	− 0.775	0	0.0411
Cell cycle	− 0.7281	0	0.0354
Non homologous end joining	− 0.6846	0.0313	0.0963
Primary bile acid biosynthesis	0.9432	0	0.006
Fatty acid metabolism	0.852	0	0.0064
Glycine serine and threonine metabolism	0.7974	0	0.0107
Retinol metabolism	0.7732	0	0.0044
valine leucine and isoleucine degradation	0.7507	0.0061	0.011
GEO cohort			
DNA replication	− 0.8487	0	0.0383
Homologous recombination	− 0.769	0	0.0293
Mismatch repair	− 0.7485	0.002	0.0435
Non homologous end joining	− 0.6924	0.002	0.0948
Cell cycle	− 0.69	0	0.0256
Primary bile acid biosynthesis	0.9223	0	0.0109
Retinol metabolism	0.8294	0.002	0.0132
Fatty acid metabolism	0.8283	0	0.0093
Glycine serine and threonine metabolism	0.8251	0.0021	0.0139

ES, Enrichment Score. Positive ES indicates enrichment in low-risk group; Negative ES indicates enrichment in high-risk group

### Tumor immunity landscape in HCC

**Fig. 4:**
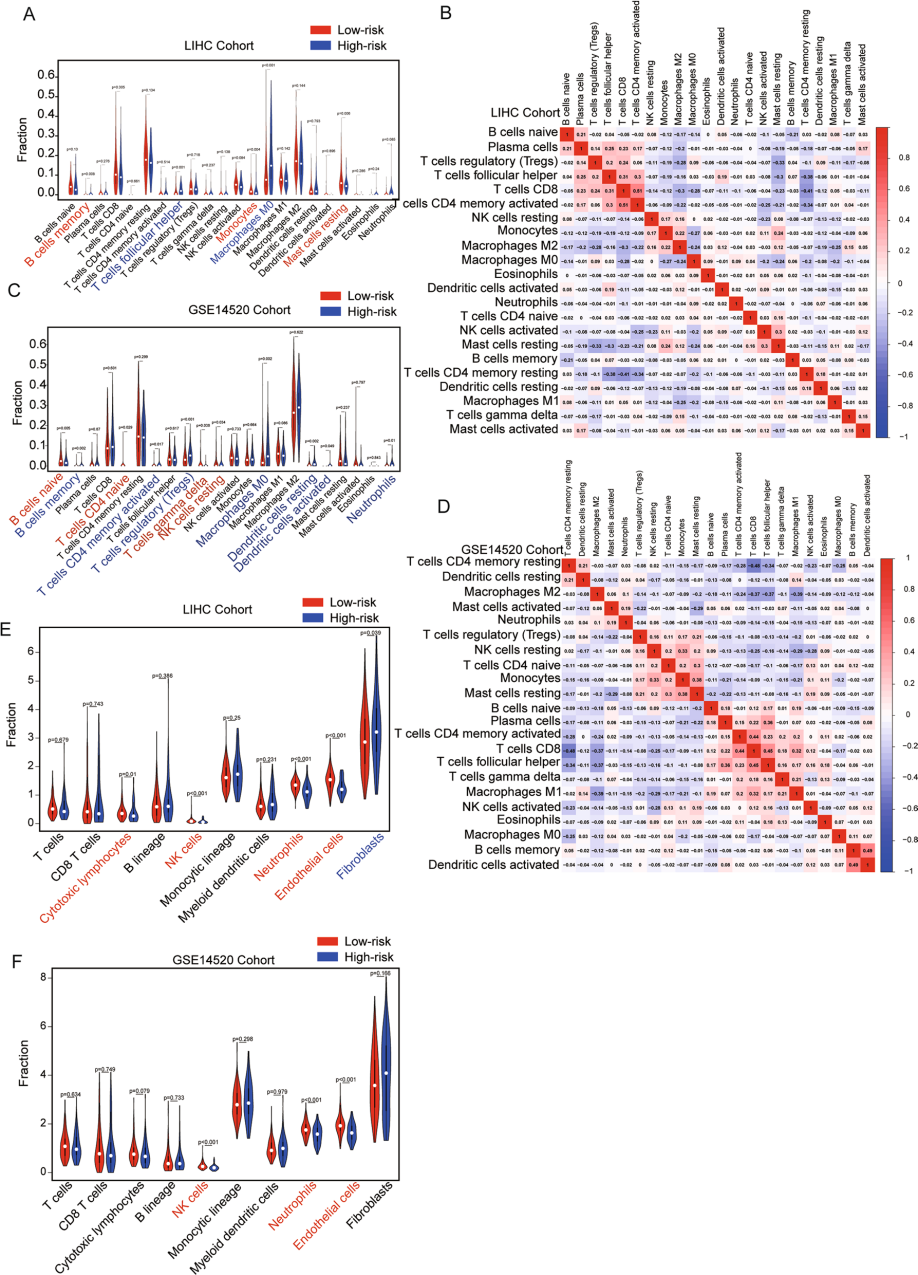
The results of immune infiltration analyses in the LIHC training cohort and GEO validation cohort. **A** Violin plot showing differences of infiltrating immune cell types between the low- and the high-risk group of CIBERSORT in TCGA cohort. **B** Correlation of risk scores and immune cell infiltration in TCGA cohort. **C** Violin plot showing differences of infiltrating immune cell types between the low- and the high-risk group of CIBERSORT in GSE14520 cohort. **D** Correlation of risk scores and immune cell infiltration in GSE14520 cohort. **E** MCP-counter show the differences of 22 types of immune cell infiltrated between the two risk groups in TCGA cohort. **F** MCP-counter show the differences of 22 types of immune cell infiltrated between the two risk groups in GSE14520 cohort

To conform whether the NHEJ-related signature was associated with immune infiltration and immunotherapy, we employed the CIBERSORT algorithm. In the TCGA cohort, the infiltration of B cells memory, T cells follicular helper and macrophages M0 were significantly higher in the high-risk group. However, in the low-risk group, monocytes and mast cells resting were more abundant (Fig. 4A). In the GSE14520 cohort, the infiltration of B cells naïve, T cells CD4 naïve, T cells gamma delta and NK cells resting were higher in the high-risk group. B cells memory, T cells CD4 memory activated, Tregs, macrophages M0, dendritic cells resting, dendritic cells activating and neutrophils were significantly more abundant in the low-risk group (Fig. 4C). We also performed correlation analysis among the 22 types of immune cells and found notable correlation between immune cells in both cohorts, such as B cells naïve and Plasma cells, CD8 T cells and macrophages M2 (Fig. 4B, D). And cytotoxic lymphocytes, NK cells, neutrophils, endothelial cells were more abundant in the low-risk group and fibroblasts were higher in the high-risk group (Fig. 4E, F). Thus, we proved that our NHEJ signature was closely related to the immune microenvironment.

**Fig. 5:**
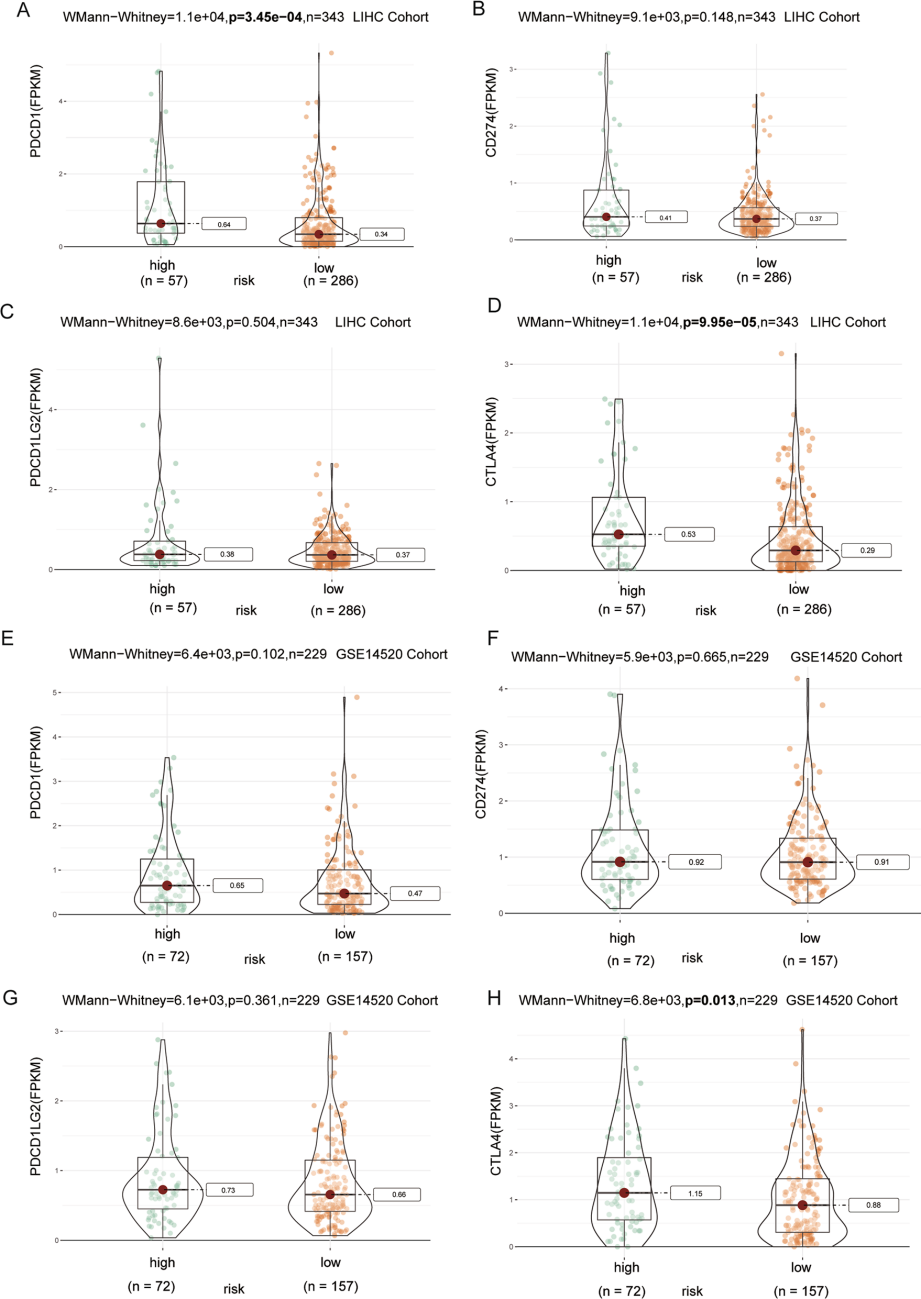
Expression of immune checkpoint molecules between the two risk groups in the TCGA training cohort and GEO validation cohort. **A**, **E*** PD-1.* **B**, **F** *CD274.* **C**, **G** *PDCD1LG2.* **D**, **H** *CTLA4*

The expression levels of four immune checkpoint genes were further investigated between the low- and high-risk groups. Compared with HCC patients in the low-risk group, patients in the high-risk group expressed higher levels of *PDCD1* and *CTLA4* (Fig. 5A–D) in TGGA cohort. Importantly, infiltrating immune cells in the tumor overexpress *PDCD1* as a strategy to evade immune responses. In GEO cohort, patients in the high-risk group expressed higher level of *CTLA4* (Fig. 5E–H). In both cohorts, the high-risk group had a higher expression level of *CTLA4*. Data from different data base-derived analyses may have the regional heterogeneity of HCC. This could account for upregulation of *PDCD1* in TCGA cohort but no difference in GEO cohort. Collectively, our results suggested an immunosuppressive landscape in HCC of high-risk group.

### Predictive nomogram construction

**Fig. 6:**
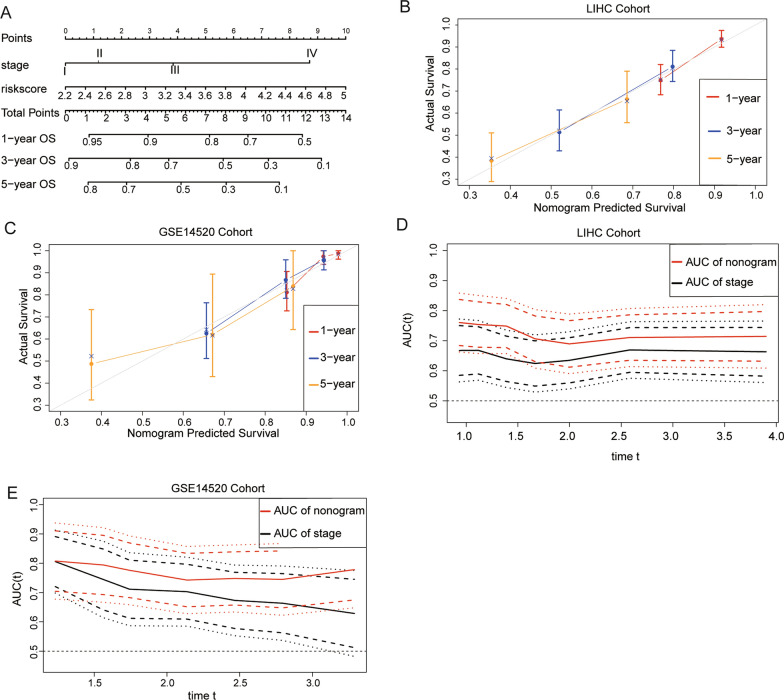
Development of a nomogram based on NHEJ genes signature for predicting OS of patients in TCGA cohort and GEO cohort. **A** The nomogram plot integrating NHEJ genes risk score, and stage. **B** The calibration plot for the probability of 1-, 3-, and 5-years OS in the TCGA training cohort. **C** Time ROC curves nomogram-based OS prediction in the TCGA training cohort. **D** The calibration plot for the probability of 1-, 3-, and 5-years OS in the GSE14520 cohort. **E** Time ROC curves nomogram-based OS prediction in the GSE14520 cohort

To construct a predictive nomogram, we performed the multivariate Cox analysis and found that the risk score of the NHEJ signature and tumor stage were independent risk factors of OS in both training cohort and validation cohort as shown in Fig. 2D and Fig. 3D. These independently associated risk factors were used to form a nomogram (Fig. 6A). The resulting model was internally validated using the bootstrap validation method. The nomogram demonstrated good accuracy, with an unadjusted C index of 0.69 (95% CI, 0.64–0.75) in the training cohort and 0.75 (95% CI, 0.68–0.82) in the validation cohort. In addition, calibration plots graphically showed good agreement between the risk estimation by the nomogram and actual survival information (Fig. 6B, C). The AUC indicated that our nomogram was more effective to predict OS of HCC patients than the sole tumor stage in both training cohort and validation cohort (Fig. 6D, E). Therefore, our risk score-based nomogram was effective to predict HCC survival.

### Deeply validation of the negative value of XRCC6 via basic exploration

**Fig. 7:**
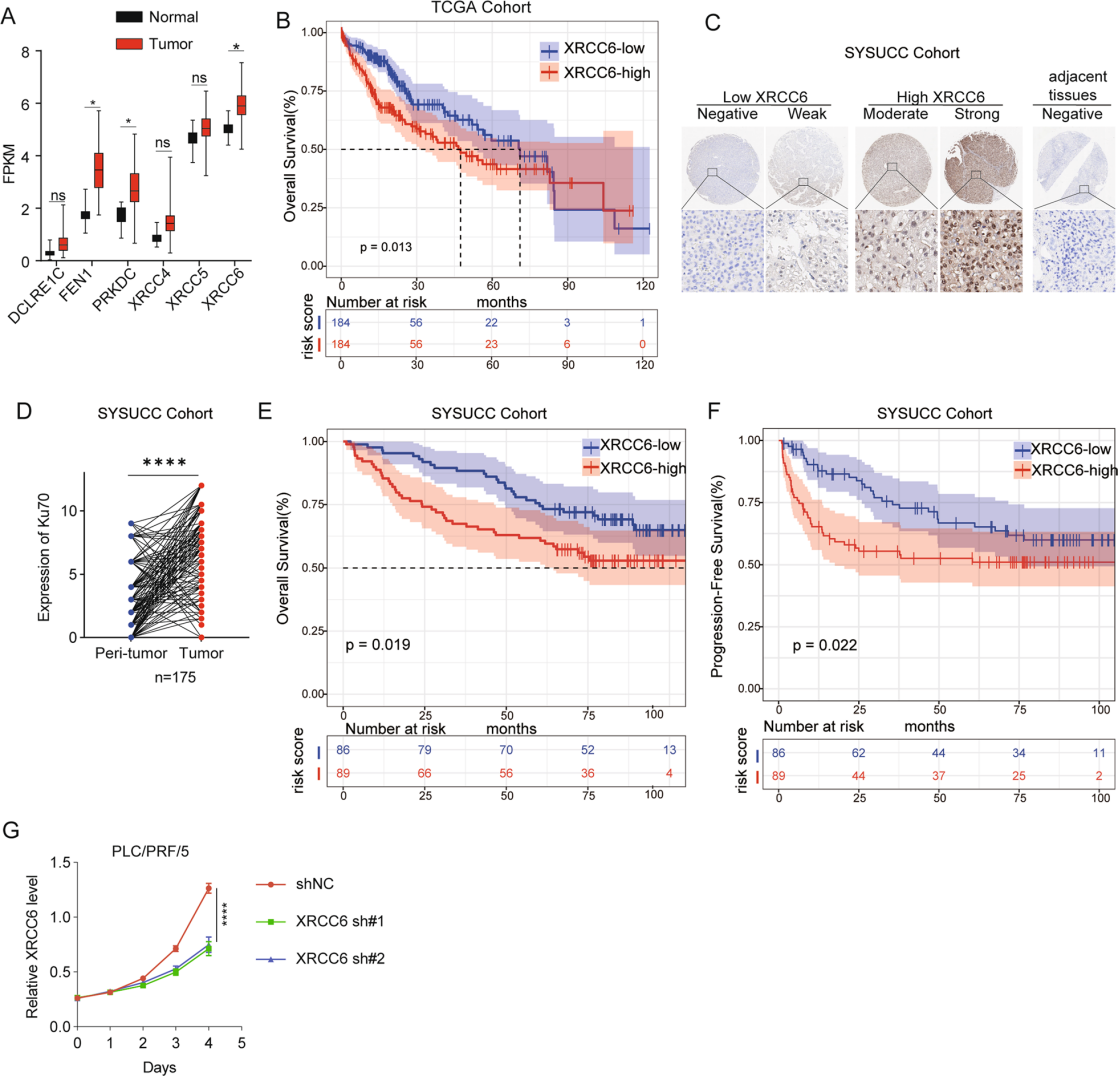
Validation of XRCC6 upregulation in HCC samples and clinical associations. **A** XRCC6 levels have the highest expression among the six NHEJ genes from TCGA RNA-seq data. **B** OS rate of HCC patients categorized according to median XRCC6 expression in TCGA cohort. **C** The representative images of IHC stain of XRCC6 in peritumor and tumor samples. **D** The IHC score of XRCC6 in paired peritumor and tumor samples (n = 175 pairs) in SYSUCC cohort. **E** OS rate of HCC patients categorized according to median XRCC6 expression in SYSUCC cohort. **F** PFS rate of HCC patients categorized according to median XRCC6 expression in SYSUCC cohort. **G** Effects of sh-XRCC6 on proliferation abilities of PLC/PRF/5 cells measured by the CCK-8 assay

Among six NHEJ genes, XRCC6 exhibited the highest expression level in TCGA RNA-seq data and was one of the core genes in NHEJ pathway. Also, XRCC6 expression was significantly elevated in HCC and elevated XRCC6 correlated with a worse OS in TCGA cohort (Fig. 7A, B). We performed IHC staining in 175 paired peritumor and tumor samples to verify the results. The representative IHC staining images of XRCC6 are shown in Fig. 7C. The protein level of XRCC6 in tumor tissues were significantly higher than paired peritumor tissues (Fig. 7D). Prognostic analysis showed that elevated *XRCC6* correlated with a worse OS and progression-free survival (PFS) in the SYSUCC (n = 175) cohort (Fig. 7E, F). The expression of *XRCC6* in HCC cells was also detected, which was up-regulated in most HCC cell lines (Additional file 1: Fig.S4A). PLC/PRF/5 cells exhibited the highest expression of *XRCC6*. Knockdown of *XRCC6* was performed to explore its role in cell proliferation (Additional file 1: Fig.S4B, C) and suppressed HCC cell proliferation was observed in *XRCC6* knockdown group (Fig. 7G).

## Discussion

Considering the increase in data-driven biological research, and ease of access to corresponding data from public databases, many studies concentrated on the relationship between RNA-seq data of specific gene sets and individual outcomes with the of numerous public databases [19]. For examples, pyroptosis-associated, platelet-related and ferroptosis-related genes were reported to predict prognosis and demonstrate immune infiltration in HCC patients [20–22]. However, there have not yet been studies of NHEJ-related genes for predicting the prognosis of HCC patients.

To clarify the relationship between NHEJ genes and the prognoses of patients with HCC, we constructed a novel prognostic risk score based on six NHEJ genes: *DCLRE1C, FEN1, PRKDC, XRCC4, XRCC5* and *XRCC6*. The risk score was used to stratify HCC patients into tow risk categories and predicted their prognosis based on the TCGA database, and then was validated in the GEO cohort. The risk score was confirmed to be an independent prognostic factor for OS according to the multivariate Cox regression analysis. Further, a predictive nomogram based on NHEJ signature was developed and validated. Moreover, we found that this NHEJ risk signature was significantly related to different antitumor immune cell infiltration levels in the tumor microenvironment of HCC.

The connection between NHEJ and DSBs is widely accepted, as a conserved pathway to repair DSBs [23]. The genomic instability is an evolving hallmark of cancer [24] and the failure of DNA repair leads to a subsequent accumulation of mutations as well as structural aberrations, usually generating particularly aggressive tumors. It’s reported that disruption of NHEJ process can drive genomic instability and accelerate the development of HCC [25]. However, failure to repair DSBs can result in increased instability and cell death through apoptosis, an essential mechanism for removing pre-cancerous cells [26]. During tumor progression or on therapy-induced tumor evolution, the DDR machinery including NHEJ pathway can be reconstituted to cope with increased replication stress and elevated levels of endogenous DNA damage [27]. In this study, we identified three NHEJ genes (*FEN1, PRKDC* and *XRCC6*) were upregulated in liver cancer tissue based on TCGA data. Besides, six of total thirteen NHEJ genes were associated with poor prognosis. Noteworthy, *PRKDC* is with 2.1% the sixth most frequently mutated DNA repair gene in all cancers and is identified as a candidate driver of hepatocarcinogenesis or therapy resistance, exhibiting frequent copy number gains [28, 29]. A large population-based study in Taiwan Province of China shows that *XRCC6* may play an important role in HCC carcinogenesis [30]. As a result of our study, GO and KEGG enrichment analysis indicated that cell cycle and DNA replication were both significantly enriched in the high-risk group. Consistently, *XRCC6* knockdown suppressed cell proliferation in vitro. Perhaps these results suggest that HCC cells become more dependent on NHEJ mechanisms to survive, proliferate and acquire resistance to treatments.

Immunotherapy of cancer has been the last major breakthrough in the fight against cancer [31]. More recently, immune checkpoint inhibition (ICI) has emerged as a first-line treatment for advanced HCC [32]. Indeed, ICIs have largely improved the prognosis of patients with intermediate and advanced HCC. However, not all patients benefit from immunotherapy and most patients would eventually experience disease progression. Thus, predictive biomarkers of ICI response are urgently needed to guide treatment decision and patient selection. The tumor microenvironment (TME) of HCC is a complex and spatially structured mixture of hepatic non-parenchymal resident cells, tumor cells, immune cells and tumor-associated fibroblasts [33]. Tumor-associated macrophages (TAMs) have a key role in cancer-related inflammation and immune response/immune escape [34]. In the present study, the high-risk group had higher proportions of M0 macrophages. While analyzed with MCP-counter algorithm, fibroblasts were higher in the high-risk group. As the most abundant components of tumor stroma, cancer-associated fibroblasts (CAFs) have been involved in the progression of liver cancer. Numerous studies have reported that CAFs promote tumor immune escape by influencing the proportion and activity of tumor immune microenvironment (TIME) [35]. NK cells play a vital role in immune monitoring to prevent the development and progression of cancer. NK cell-based anti-HCC therapeutic approaches are becoming increasingly attractive [36]. We also observed that infiltrating proportions of NK cells were apparently higher in low-risk patients. Moreover, the high-risk group had a higher expression level of *CTLA4*. Taken together, these results revealed an immunosuppressive TME in high-risk group patients. Therefore, our results suggest that the risk score could provide a basis for immunotherapy to screen patients who respond to ICI treatment.

There are several limitations in our study. Firstly, we found that NHEJ-based risk model was closely related to the TIME of HCC patients. However, we failed to include and analyze immunotherapy cohorts to explore whether the model could predict its efficacy. Secondly, although the immune cell composition was calculated based on various algorithm, it is still inaccurate compared with IHC and flow cytometry. Thirdly, we proved the function of *XRCC6* in HCC cells, the other NHEJ factors should also be verified in HCC. Lastly, the biological function of *XRCC6* was just verified in vitro, further animal experiments should be performed in the future.

In conclusion, we constructed a risk signature and nomogram to predict prognosis and tumor immune infiltration of HCC in two independent cohorts with high accuracy. The NHEJ risk model has the potential to be used as a biomarker to develop more individualized treatment for HCC patients.

## Supplementary Information

**Additional file 1: Methods S1.** **Table S1.** NHEJ gene set from MSigDB (http://www.gsea-msigdb.org). **Table S2.** Antibodies included in the study. **Table S3.** The primers and shRNA used in present study. **Figure S1.** Flow chart of data collection and analysis. **Figure S2.** Assessment of prognostic value of the NHEJ signature model in the TCGA and GEO cohort. **Figure S3.** Gene set enrichment analysis between the high- and low-risk subgroups in TCGA training cohort and the GEO validation cohort. **Figure S4.** Validation of XRCC6 upregulation in HCC samples and clinical associations.

**Additional file 2: Table S4.** Gene set enrichment analysis between the high- and low-risk subgroups in TCGA training cohort.

**Additional file 3:** **Table S5.** Gene set enrichment analysis between the high- and low-risk subgroups in GEO validation cohort.

## Data Availability

Publicly available datasets were analyzed in this study. This data can be found here: TCGA databases (https://tcga-data.nci.nih.gov/tcga/) and the GEO databases (https://www.ncbi.nlm.nih.gov/geo/). In accordance with the journal’s guidelines, the data presented in this study are available on request from the corresponding author.
