# Supplementary material for: A novel NHEJ gene signature based model for risk stratification and prognosis prediction in hepatocellular carcinoma
Source: Cancer Cell Int. 2023 Apr 4;23:59. doi: 10.1186/s12935-023-02907-9 (PMC10071660; doi:10.1186/s12935-023-02907-9)
Supplement: Supplementary file 1 — Additional file 1: Methods S1. Table S1. NHEJ gene set from MSigDB (http://www.gsea-msigdb.org). Table S2. Antibodies included in the study. Table S3. The primers and shRNA used in present study. Figure S1. Flow chart of data collection and analysis. Figure S2. Assessment of prognostic value of the NHEJ signature model in the TCGA and GEO cohort. Figure S3. Gene set enrichment analysis between the high- and low-risk subgroups in TCGA training cohort and the GEO validation cohort. Figure S4. Validation of XRCC6 upregulation in HCC samples and clinical associations. [file 12935_2023_2907_MOESM1_ESM.docx]

**Supplementary Methods, Tables and Figures**

**Supplemental Methods**

**Cell Lines and Cultivation Condition**

Human HCC cell lines (PLC/PRF/5, Huh7, Hep3B, MHCC-97H, MIHA, HCC-LM3，JHH-7 and HepG2) were purchased from Shanghai Cell Bank of the Chinese Academy of Sciences (Shanghai, China) with STR (short tandem repeat) appraisal certificates. At 37°C and 5% CO_2_, cells were grown in Dulbecco's Modified Eagle medium with 10% fetal bovine serum.

**Immunohistochemical staining (IHC)**

As previously described, IHC staining was performed(1). Paraffin-embedded tissue sections and tissue arrays were incubated with anti-XRCC6 antibody overnight at 4 °C followed by respective secondary antibodies. Staining intensity was measured as follows: negative = 0, weak = 1, moderate = 2, or strong = 3. The percentage of positive tumor cells was assessed as follows: 0 (0–10% positive cells), 1 (10–25%), 2 (25–50%), 3 (50–75%) and 4 (>75%).

**RNA extraction and quantitative real-time PCR**

As previously described, RNA extraction was performed(1). The reverse-transcribed cDNA products were used for qPCR analysis using SYBR Green PCR kit (Invitrogen, California, USA).

**Western blotting assays**

The extracted whole cell protein lysates were prepared with cell lysis buffer (Cell Signaling Technology, Boston, USA) supplemented with protease inhibitor cocktail (Roche, Basel, Switzerland). Then, the equal amounts of protein lysates were separated by SDS-polyacrylamide gels and transferred to polyvinylidene difluoride membranes (Merck Millipore, Cork, Ireland). After incubated with the indicated anti-XRCC6 and secondary antibodies the bindings were detected using High-sig ECL western blotting kit (Tanon, shanghai, China).

**Cell proliferation assays**

The cell proliferation was conducted with Cell Counting kit-8 (CCK-8; DojinDo, Japan) reagent. Briefly, 1×10^3^ cells were seeded in 96-well plates and cultured in DMEM (Dulbecco's Modified Eagle Medium) containing 10% FBS. CCK8 reagent, at a final concentration of 10%, was then added to each well for 2h at 37 °C. The OD value was measured at 450 nm in a microplate reader epoch2 (Bio-Tek, USA). Six repeated wells were used for each experimental condition, and three independent experiments were performed.

**Cell Transfection**

shRNAs were synthesized and obtained from the DesignGene Biotechnology (Guangzhou, China). HCC cells in well condition were uniformly seeded into 6-well plates in advance, and transfection was performed with Lipofectamine 2000 (Invitrogen, CA, USA) following the manufacturer’s instruction when the cells were adherent and reached 70% confluence.

**Detailed antibody, primers and shRNAs lists and application is supplied as Table S2 and Table S3.**

**Table S1. NHEJ gene set from MSigDB(**[**http://www.gsea-msigdb.org**](http://www.gsea-msigdb.org)**).**

| **Gene Symbol** | **Full Name** |
| --- | --- |
| *RAD50* | RAD50 double strand break repair protein |
| *DNTT* | DNA nucleotidylexotransferase |
| *FEN1* | flap structure-specific endonuclease 1 |
| *XRCC6* | X-ray repair cross complementing 6 |
| *POLL* | DNA polymerase lambda |
| *POLM* | DNA polymerase mu |
| *LIG4* | DNA ligase 4 |
| *MRE11* | MRE11 homolog, double strand break repair nuclease |
| *PRKDC* | protein kinase, DNA-activated, catalytic subunit |
| *DCLRE1C* | DNA cross-link repair 1C |
| *XRCC4* | X-ray repair cross complementing 4 |
| *XRCC5* | X-ray repair cross complementing 5 |
| *NHEJ1* | non-homologous end joining factor 1 |

**Table S2. Antibody included in the study.**

| **Antibodies** | **SOURCE** | **IDENTIFIER** |
| --- | --- | --- |
| Anti-XRCC6 (for WB, IHC)  1:1000 for WB  1:200 for IHC | 10723-1-AP | Proteintech Group, Chicago, IL, USA |

**Table S3. The primers and shRNA used in present study.**

| **Primer names** | **Forward (5’-3’)** | **Reverse (5’-3’)** |
| --- | --- | --- |
| *XRCC6* | GAGAAACAGGATGTGAGAAATCG | TCTGCTTCATTATCTCACGCTCT |
| **shRNA** | | |
| shXRCC6#1 | GATGAGTCATAAGAGGATCAT | ATGATCCTCTTATGACTCATC |
| shXRCC6#2 | CGTCAGATTATACTGGAGAAA | TTTCTCCAGTATAATCTGACG |
| shNC | CGGGGCGAGGAGCTGTTCAC | ACGUGACACGUUCGGAGAATT |

**Fig.S1 Flow chart of data collection and analysis.**


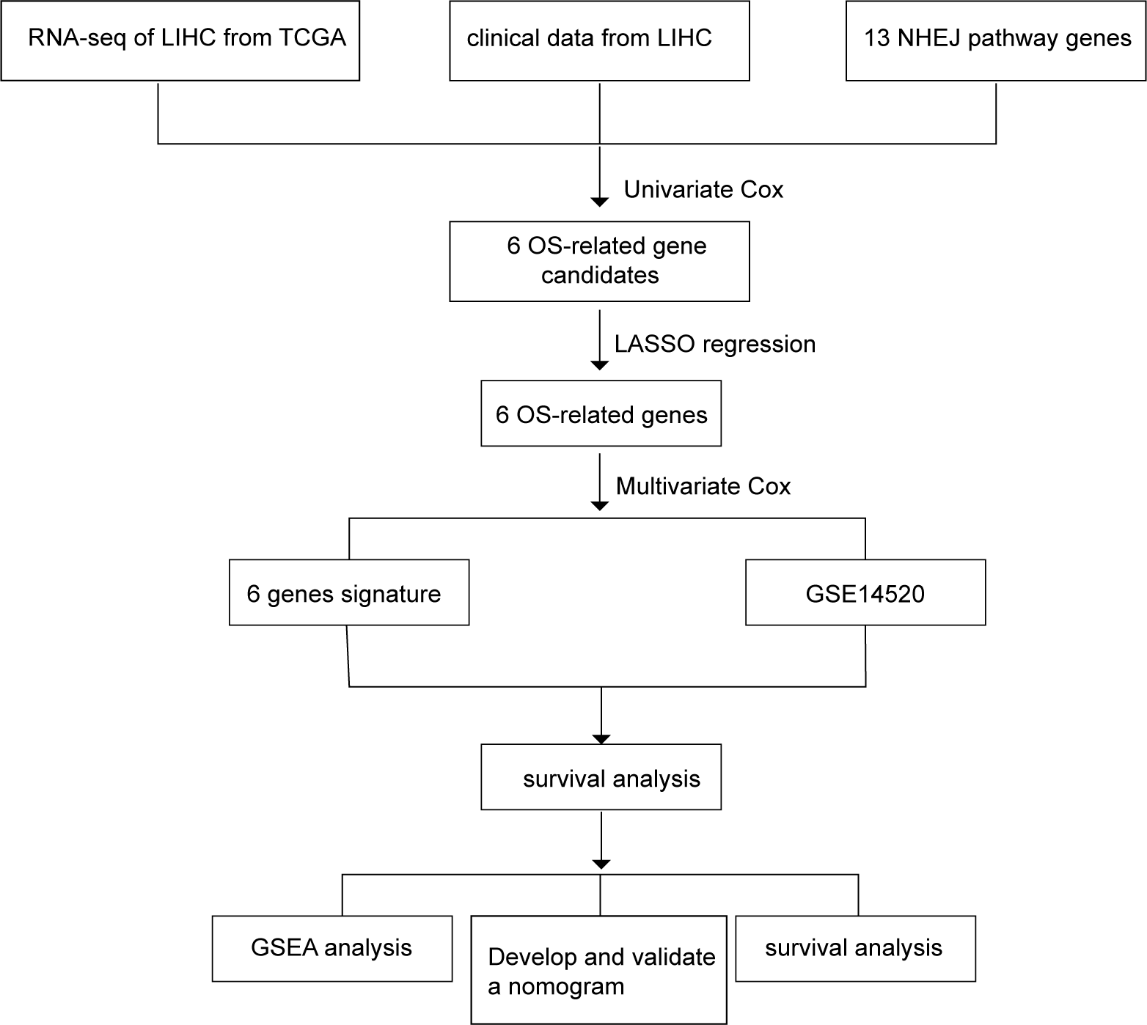


**Fig.S2 Assessment of prognostic value of the NHEJ signature model in the TCGA and GEO cohort.**


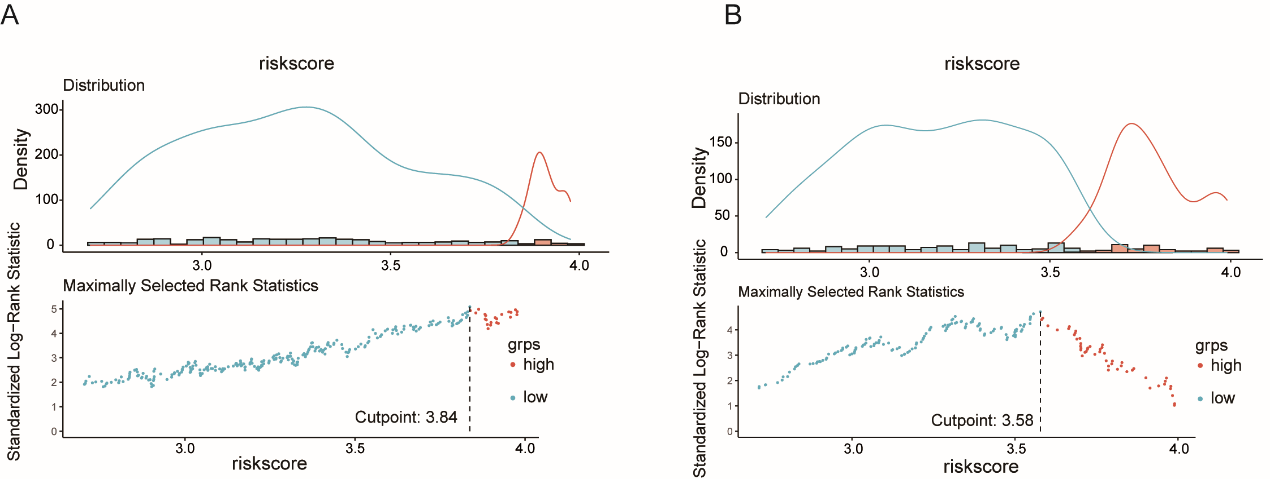


1. Determination of cut-off value of NHEJ risk scores by the maximally selected log-rank statistics in LIHC cohort.
2. Determination of cut-off value of NHEJ risk scores by the maximally selected log-rank statistics in GEO cohort.

**Fig.S3 Gene set enrichment analysis between the high- and low-risk subgroups in TCGA training cohort and the GEO validation cohort.**


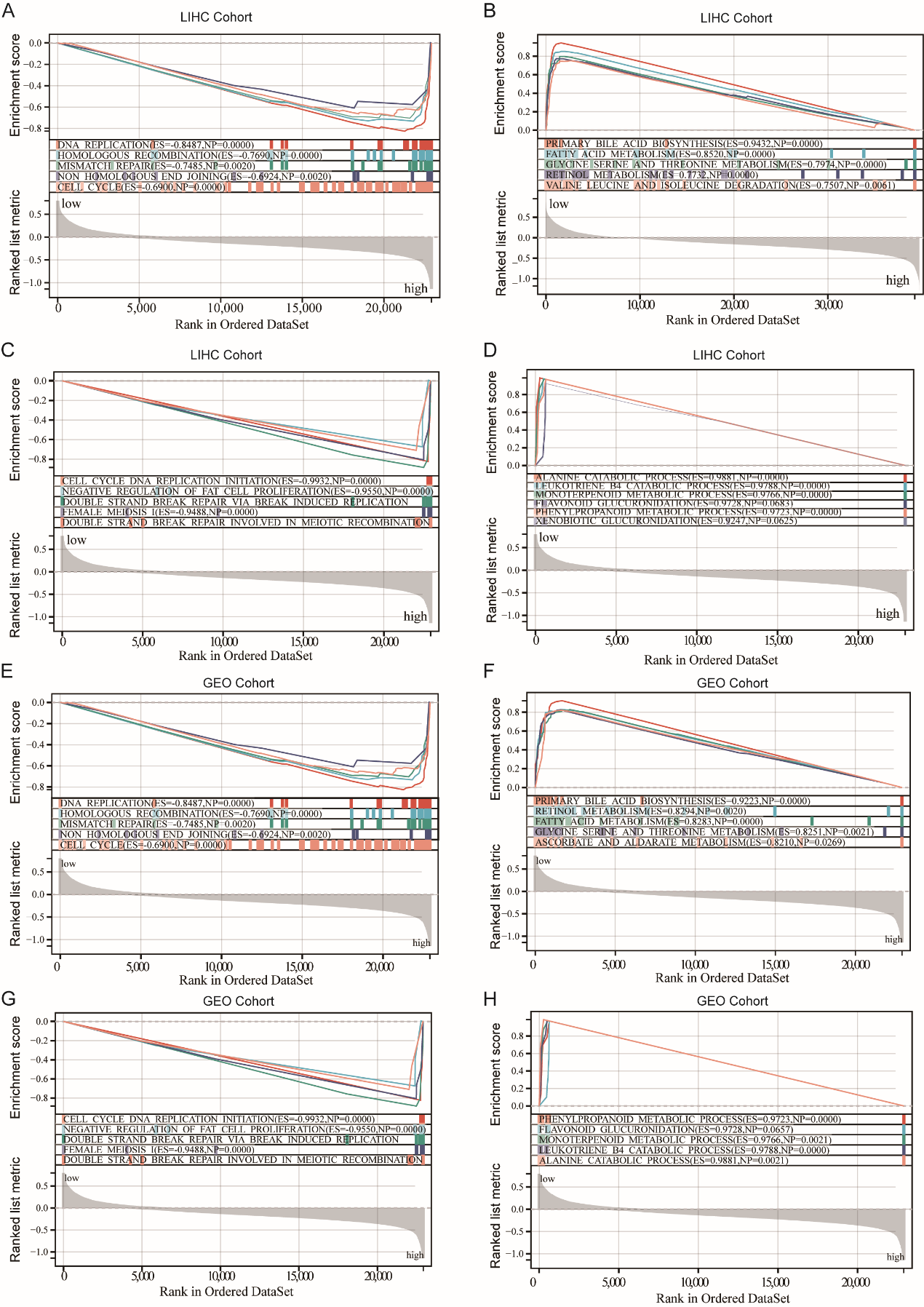


1. (B) Enriched KEGG terms between high- and low-risk groups in TCGA cohort. NP, normalized p value.
2. (D) Enriched GO pathways between high- and low-risk groups in TCGA cohort.
3. (F) Enriched KEGG terms between high- and low-risk groups in GEO cohort.
4. (H) Enriched GO pathways between high- and low-risk groups in GEO cohort.

**Fig.S4 Validation of XRCC6 upregulation in HCC samples and clinical associations.**


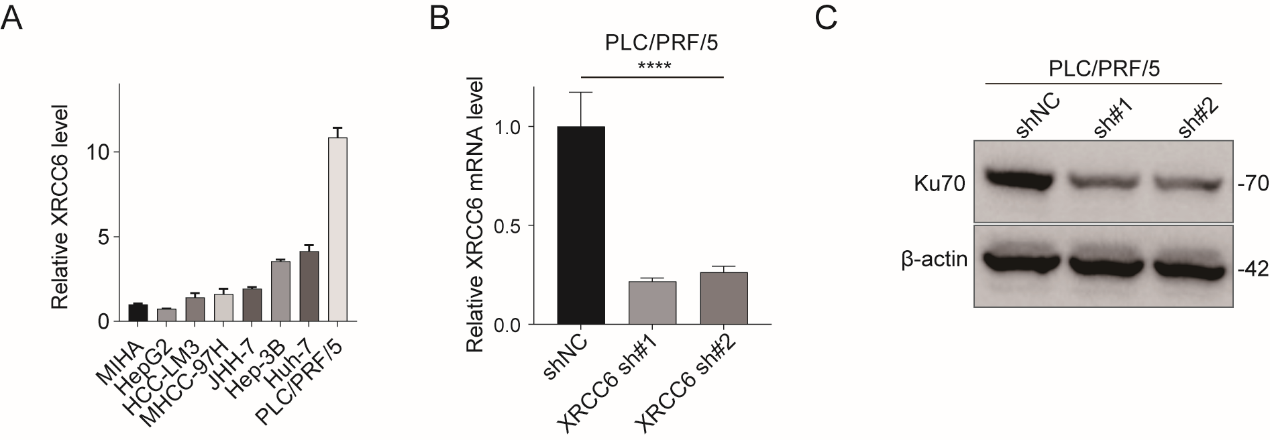


1. The expression of *XRCC6* in different HCC cell lines.
2. Q-PCR validation of *XRCC6* knockdown in PLC/PRF/5 cells.
3. Western blot validation of *XRCC6* knockdown in PLC/PRF/5 cells.

**Reference**

1.Zhong C, Niu Y, Liu W, Yuan Y, Li K, Shi Y, Qiu Z, et al. S100A9 Derived from Chemoembolization-Induced Hypoxia Governs Mitochondrial Function in Hepatocellular Carcinoma Progression. Adv Sci (Weinh) 2022:e2202206.
